# 

**Published:** 1891-12

**Authors:** 


### CONTENTS

### *EDITORIALS.*

| | PAGE |
|---|---|
| A Retrospect, . . . . . . . . . . . . . . | 441 |
| Is it Homœopathy or Isopathy? . . . . . . . . | 443 |

### *MISCELLANEOUS.*

| | |
|---|---|
| Is it Homœopathy or Isopathy? Samuel Swan, M. D., . . . . | 444 |
| In Memoriam—P. P. Wells, M. D., . . . . . . . . . . | 444 |
| What are the Remedies? Robert Farley, M. D., . . . . . | 448 |
| Provings and Clinical Observations with High Potencies. Malcolm Macfarlan, M. D., . . . . . . . . . . . . | 449 |
| Science and Old Medicine Contrasted. T. F. Pomeroy, M. D., . . | 457 |
| Chronic Intoxication from the Habitual Use of the Essences, as Wermuth, Absinthe, etc. S. L., . . . . . . . . . | 466 |
| British Medicinal Plants. Alfred Heath, M. D., F. L. S., . . . | 468 |
| Cholera Infantum. Wm. Steinrauf, M. D., . . . . . . | 470 |
| In Memoriam—Samuel Lilienthal, M. D., . . . . . . | 473 |

### *BOOK NOTICES.*

| | |
|---|---|
| Our Animal Friends. . . . . . . . . . . . . | 474 |
| On the Powers of Absorption of Mucous Membrane of Urinary Bladder. Dr. B. London, . . . . . . . . | 474 |
| The Clinical Guide. By G. H. G. Jahr, . . . . . . . . | 474 |
| Scientific Medicine in its Relation to Homœopathy. Prof. Bakody, . | 475 |
| The Cheltenham Reveille, . . . . . . . . . . | 475 |
| Annals of Surgery, . . . . . . . . . . . . . | 475 |
| A Treatise on Practical Anatomy. By Henry C. Bœnning, M. D. . | 476 |
| The Greater Diseases of the Liver. By J. Compton Burnett, M. D., . | 476 |

### *NOTES AND NOTICES.*

| | |
|---|---|
| Dr. M. A. A. Wolff—Removals—Dr. Rufus Choate—Dr. Harriet H. Cobb—Dr. George A. Taber—*Printers' Ink*—College of Physicians and Surgeons—Fincke's Translation of *The Organon*—Lacto-Cereal Food—Wells on Intermittent Fever—The Ballard Binding Klip—The Bates Numbering Machine—The Cleveland Homœopathic Hospital College—Dr. Bushrod W. James—The National Homœopathic Medical College of Chicago—A Private Homœopathic Insane Asylum—The Cleveland Medical College—Orificial Surgery—Kansas City Homœopathic Medical Society—Missouri Institute of Homœopathy—The Texas State Society—Illinois State Society—Long Island College Hospital—Ohio State Society, . . . . . . . . . . . . . | 476 |

